# Osmotically Enabled Wearable Patch for Sweat Harvesting and Lactate Quantification

**DOI:** 10.3390/mi12121513

**Published:** 2021-12-04

**Authors:** Tamoghna Saha, Jennifer Fang, Sneha Mukherjee, Charles T. Knisely, Michael D. Dickey, Orlin D. Velev

**Affiliations:** Department of Chemical and Biomolecular Engineering, North Carolina State University, Raleigh, NC 27695-7905, USA; tsaha@ncsu.edu (T.S.); jfang7@ncsu.edu (J.F.); smukhe22@ncsu.edu (S.M.); ctknisel@ncsu.edu (C.T.K.)

**Keywords:** paper microfluidics, sweat, sensing, hydrogels, lactate, osmotic pumping, evaporation, capillary, wicking, biochemical assay

## Abstract

Lactate is an essential biomarker for determining the health of the muscles and oxidative stress levels in the human body. However, most of the currently available sweat lactate monitoring devices require external power, cannot measure lactate under low sweat rates (such as in humans at rest), and do not provide adequate information about the relationship between sweat and blood lactate levels. Here, we discuss the on-skin operation of our recently developed wearable sweat sampling patch. The patch combines osmosis (using hydrogel discs) and capillary action (using paper microfluidic channel) for long-term sweat withdrawal and management. When subjects are at rest, the hydrogel disc can withdraw fluid from the skin via osmosis and deliver it to the paper. The lactate amount in the fluid is determined using a colorimetric assay. During active sweating (e.g., exercise), the paper can harvest sweat even in the absence of the hydrogel patch. The captured fluid contains lactate, which we quantify using a colorimetric assay. The measurements show the that the total number of moles of lactate in sweat is correlated to sweat rate. Lactate concentrations in sweat and blood correlate well only during high-intensity exercise. Hence, sweat appears to be a suitable biofluid for lactate quantification. Overall, this wearable patch holds the potential of providing a comprehensive analysis of sweat lactate trends in the human body.

## 1. Introduction

Lactate is a by-product of glycolysis, which is generated as an outcome of anaerobic glucose metabolism due to the high energy demand of the body. It is a pivotal biomarker for determining oxidative stress levels, muscle health, and tissue hypoxia [1,2,3,4]. Lactate is predominantly present in eccrine sweat [5], blood [3,6], and to a certain extent in tears [7] and saliva [8]. The net amount of lactate present in these biofluids is highly dependent on the physiological state and the site of anaerobic metabolism in the human body. Lactate monitoring is of prime importance in certain groups of individuals, especially those who are frequently prone to oxygen-deficit conditions such as athletes and military personnel [3] (for improving training and performance endurance). Critical lactate levels in the body can abruptly alter the fluid pH and cause several detrimental effects on human health such as seizures, sepsis, renal failure, tumors [9], cerebral stroke [10], and panic disorders [11].

Lactate content in eccrine sweat depends on several parameters such as the exertion type, duration, intensity, and sweat rate [11]. Medium-intensity exercise (60–70% of maximum heart rate) for a short duration, or heat-induced sweating can generate a lactate concentration of up to 25 mM, while exhaustive outdoor exercises could generate up to 115 mM [12,13,14,15,16]. On a contrary note, it is also proven that the sweat lactate concentration varies inversely with sweat rate [17]. This suggests that with rigorous physical activity, the rate of sweat release is greater than the rate of lactate generation. There is also evidence that suggests sweat lactate levels vary minimally with age [15], sex [15,18], and the sampling location (due to varying sweat gland density) [6,19]. Apart from eccrine sweat glands, there two other types of sweat glands in the body have been identified: apocrine and apoeccrine [5]. However, there is less evidence of lactate being present in the sweat secreted by these glands, and our focus here is on eccrine glands in the forearms as a commonly accepted lactate testing location.

Similar to most other biomarkers, researchers have looked upon the correlation between the lactate concentration in sweat and blood. Alam et al. have evaluated that blood lactate concentration ranges between 0.5 and 2 mM for a healthy subject at rest [3]. During a low to medium-intensity exercise workout, the blood lactate concentration neither shows a significant change in its magnitude nor reflects a good correlation with sweat lactate concentration [13,20]. Goodwin et al. have shown that once the body crosses its lactate threshold (LT), blood lactate concentration rises exponentially and can peak up to 11 mM [21]. Such high blood lactate can inhibit muscle contractions and is undesirable. A study by Karpova et al. has shown a positive correlation between blood and sweat lactate levels during high-intensity exercise [1]. Thus, the extent of lactate generation in both sweat and blood are well understood under active sweating. However, to the best of our knowledge, there are no literature reports of studies that simultaneously monitor sweat and blood lactate levels under low sweating conditions or after a sufficiently long-time post exercise.

Researchers have been able to quantify sweat and blood lactate levels using different sensing and quantification techniques [7,11,12,13,14,15,16,17,18,19,20,21,22,23,24,25,26,27,28,29,30,31,32,33,34,35,36,37,38,39,40,41,42,43,44,45,46,47,48,49,50,51,52,53,54]. Established commercial and laboratory-based sensing platforms function in the presence of an enzyme (either lactate oxidase (LO_x_) or lactate dehydrogenase (LDH)). Earlier skin-interfacing wearable sensor prototypes developed on colorimetric principles typically operate by transporting sweat through complex serpentine microfluidic channels to a sensing zone, which contains a lactate specific enzymatic assay. The incoming lactate in the sweat reacts and generates a colored product whose intensity is quantified via phone-based image analysis. This intensity directly correlates to the concentration of lactate in sweat [22,23,24,25].

Another class of wearable device prototypes involve electrochemical measurements where lactate concentration is determined based on the magnitude of the current generated at the functionalized electrode surface [26,27,28,29,30,31,32,33,34,35,36,37,38,39,40,41,42,43,44,45,46,47,48,49,50,51]. Such sensing platforms usually comprise a three-electrode system where the enzyme is immobilized on the working electrode either via cross-linking, adsorption, electrostatics, or covalent bonding [3]. Lactate in sweat can be also quantified using chemiluminescence [52,53], HPLC [30,55], or enzyme free colorimetry [47].

Despite delivering on-spot sweat lactate detection with high sensitivity, wide linear dynamic range (LDR), and low limit of detection (LOD), the current wearable platforms face the following three key challenges: they (1) remain inoperative under the conditions of normal human activity—which rarely involves profuse sweating [7,8,12,22,23,24,25,40,41,42,44,48,52]; (2) do not provide a comprehensive analysis of the human lactate metabolism and its correlation to blood lactate levels [27,30,34,36,40,41,42,46,47,48,50,56]; and (3) require an external power source for continuous operation [8,12,36,40,41,42,47,48,50,51,52,54]. Thus, most of the current sweat lactate sensors function only under “active sweating”; i.e., they either require the subjects to undergo strenuous physical exertion, ingest sweat stimulating agents [36,57], or elevate body temperature [13] prior to testing. As a result, these prototypes can quantify the lactate concentration in sweat only during exercise and not under any other physiological condition (such as rest or post-exercising conditions). Furthermore, conducting measurements via active sweating is not only inconvenient and tedious but also raises questions concerning its clean capture, external contamination, low sweat volumes due to uncontrolled evaporation, and dilution of biomarkers because of excessive sweating. Some prototypes have quantified sweat lactate release from rested subjects [34,36,58]. However, these prototypes are not a wearable platform, have a low sampling duration, do not report their sweat lactate results post-exercise, and do not study the correlation between blood and sweat lactate levels. Hence, there is a necessity of an efficient sweat lactate monitoring device, which is capable of delivering information under all physiological conditions (rest, during exercise, and post-exercise), and providing a comprehensive understanding of the human lactate metabolism remains unaddressed.

This article discusses the on-skin performance of our recently developed zero-powered, non-invasive wearable sweat sampling patch [14,59,60]. We use three different pumping materials (based on different osmotic strength) in our patch for sampling sweat lactate from the forearm region of the body. We chose the forearm, since it is standard for such measurements and has one of the highest sweat gland densities in the body [5]. The patches were tested under five different physiological conditions (rest, medium-intensity exercise, high-intensity exercise, post-medium-intensity exercise, and post-high-intensity exercise). We initially present a comparative analysis of the amount of lactate sampled by each pumping source. Then, we discuss the operating mechanism of each osmotic on-skin pumping material toward facilitating long-term lactate withdrawal and estimate how much lactate is sampled via osmosis. Next, we study the dependency of sweat lactate on the sweat rate. Finally, we seek to understand the correlation between sweat and blood lactate levels.

## 2. Experimental Section

### 2.1. Materials

The experiments were performed using Whatman filter paper (Grade 542, GE Healthcare Life Sciences, Waukesha, WI, USA); Sylgard-184 elastomer (Dow Corning, Midland, MI, USA); phosphate buffer saline (PBS), acrylamide (Sigma, St. Louis, MO, USA), N-N′ methylenebisacrylamide (Sigma, St. Louis, MO, USA), 2-hydroxy-4′-(2-hydroxyethoxy)-2-methylpropiophenone (Sigma, St. Louis, MO, USA), D-glucose, alcohol wipes (medical grade), L-lactic acid quick test strips (QQLLAC10, BioAssay Systems, Hayward, CA, USA), and a lactate plus meter (Nova Biomedical, Waltham, MA, USA).

### 2.2. Patch Fabrication

Sylgard-184 silicone elastomer and its curing agent were mixed in a 10:1 *w*/*w* ratio and cured for 12 h at 70 °Cto make the base for the PDMS sheet. The patch was prepared by attaching two PDMS sheets together—38 mm × 15 mm × 2 mm (bottom) and 30 mm × 15 mm × 1 mm (top). A single hole matching the hydrogel diameter was punched at 6 mm from one of the edges on the bottom sheet to encase the hydrogel. A section of Whatman 542 paper was cut out using a CO_2_ laser cutter (Universal Laser Systems VLS 3.5, Scottsdale, AZ, USA) and was sandwiched between the top and bottom PDMS sheets. Our previous research on capillary-assisted evaporative pumping determined the dimension of the paper channel for this study [59]. The PDMS sheets were attached together using additional silicone as a binder (Sylgard-184), making sure that it did not contact the paper channel. The whole patch was treated in an oven at 40 °C overnight to achieve firm adhesion between the sheets.

### 2.3. Hydrogel Synthesis and PDMS Disk Fabrication

The hydrogels for the osmotic interface were made using acrylamide monomer, N, N′-methylenebisacrylamide as the crosslinker and 2-hydroxy-4′-(2-hydroxyethoxy)-2-methylpropiophenone as the photo-initiator. The monomer solution contained 22% (*w*/*w*) acrylamide, 0.48% (*w*/*w*) crosslinker, and 0.15% (*w*/*w*) photo-initiator. The solution was cured inside a circular Petri dish (47 mm diameter) under a 175 mW/cm^2^ UV lamp (Sunray 400-SM, Uvitron International, West Springfield, MA, USA) for three minutes. Disks of 6 mm diameter were punched out and stored in either 4 M glucose or PBS solution for 24 h. These solutions served as the osmolytes. After 24 h, the infused disks were transferred to a fresh solution in a vial and stored for further usage. A single disk was taken out, blotted with a paper napkin (to remove the residual osmolyte on the hydrogel surface), and fitted inside the circular space in the patch before usage.

Disks of 6 mm diameter were punched out from a 2 mm thick PDMS sheet. This was referred as the PDMS disk. The PDMS disk was fitted inside the circular space in the patch before usage.

### 2.4. On-Skin (In Vivo) Sweat Analysis

Human trials were conducted on individuals in five sets: (a) NEX (i.e., without exposing them to any physical activity) for 2 h; (b) during MEX for an hour; (c) PMEX for 2 h; (d) during HEX for 30 min; and (e) PHEX for 2 h. All trials were conducted under normal ambient conditions (22 °C, 45% relative humidity (RH)) and strictly as per two approved IRB protocols (UNC-18-1959 and UNC-19-3065). Eight healthy subjects (5 females and 3 males), aged 20–28, were recruited from the NC State University campus. All 8 subjects were tested under NEX, MEX, and PMEX trials, while 3 subjects were also tested under MEX and PMEX trials. All subjects gave written, informed consent before participation in the study.

We used three types of patches for our human trials, which differ by the type of their pumping material: 4 M glucose hydrogel, PBS hydrogel, and PDMS disk. Both subjects’ forearms were initially washed with alcohol and DI water. Then, these patches were snapped on both forearms of every individual using a Velcro strap. The subjects resided on a bench for 2 h when testing under NEX. After 2 h, the patches were taken off and stored for analysis. A paper strip was also gently rubbed (dry rub test) on the skin to check the base lactate level after 2 h. Blood lactate was measured using the conventional finger-pricking test. This concluded the NEX trials. The skin was rewashed using alcohol and DI water, and a fresh new batch of all three patch types was interfaced onto the skin again during the MEX trials. After an hour, the patches were taken off, and a dry rub test was performed. Blood lactate was also measured. This concluded the MEX trials. The skin was washed again with alcohol and DI water. A fresh new batch of all three patch types was interfaced onto the skin again for 2 h. After 2 h, the patches were taken off and stored for analysis. Additionally, a dry rub test and blood lactate test were also conducted. This concluded the PMEX trials.

The HEX and PHEX trials were conducted 24 h post PMEX trial completion. The skin was initially washed with alcohol and DI water. Then, the patches were snapped on both forearms. The subjects exercised under HEX for 30 min. After the test, both dry rub and blood lactate tests were conducted. The protocol followed for the PHEX trial was the same as that for the PMEX trial. The subjects refrained from any food or drinks throughout the testing period. The paper channel in the patch was treated with our previously developed colorimetric lactate assay (lactate dehydrogenase based) to determine the lactate levels after the completion of each trial [14]. A further detailed description of the testing protocol is provided in the Supplementary Information.

### 2.5. Statistical Analysis

We performed a two-sample t-test for statistical significance. Significance is denoted as *p* < 0.05.

## 3. Results and Discussion

### 3.1. Sweat Sampling Mechanism of the Wearable Patch

**Figure 1:**
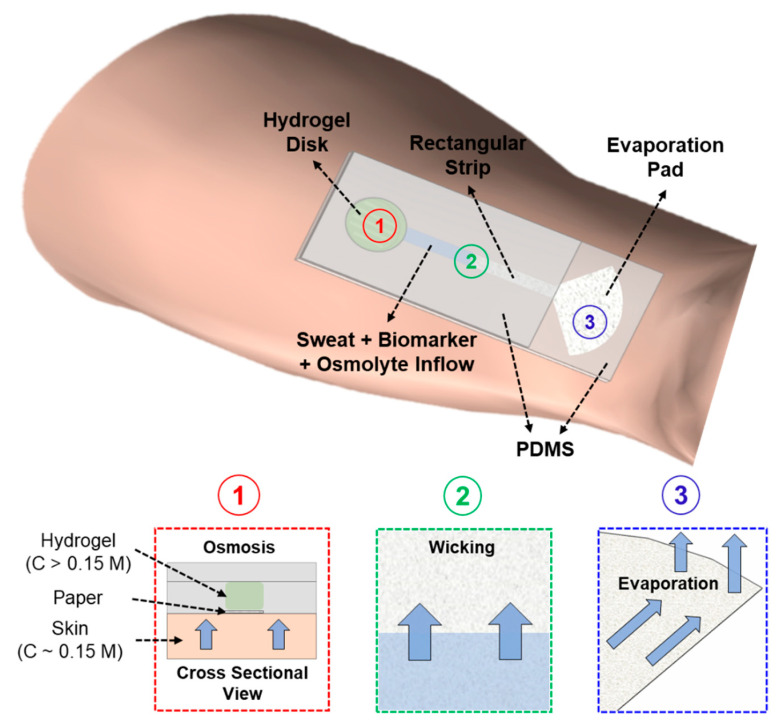
Schematic of the wearable, zero-powered, and non-invasive sweat sensing platform on skin. The platform comprises of a hydrogel pad and a paper microfluidic channel. Simultaneous action of osmosis, capillary wicking, and evaporation leads to long-term sweat withdrawal from skin even at resting perspiration rates.

Our wearable patch functions based on the simultaneous action of three basic effects to deliver long-term sweat and biomarker sampling: osmosis, capillary wicking, and evaporation [14]. Osmosis is the main pumping mechanism for sweat extraction. A hydrogel infused with a highly concentrated solute (osmolyte) generates the osmotic driving force with the skin. The hydrogel is hosted inside the circular chamber of the patch, as shown in Figure 1. The hydrogel disk and the circular end of the paper directly interface the skin. The hydrogel withdraws sweat and its associated biomarkers due to its higher osmotic strength (with respect to sweat) (Figure 1). These withdrawn sweat and biomarkers are sampled on a paper microfluidic channel. The paper channel has two sections: (a) a long rectangular strip (sandwiched between two PDMS sheets), with one end in contact with the hydrogel and the other end connected to (b) a pad with a large surface area (evaporation pad), which is left open to allow sweat evaporation. The sweat, biomarkers, and the osmolyte travel through the rectangular section of the paper channel via capillary action toward the evaporation pad. The incoming sweat evaporates from the pad, leaving behind all biomarkers and non-volatile osmolytes. These biomarkers can be quantified using different analytical techniques. Since osmosis is a colligative property, the patch does not require any external power source for long-term operation. The patch will continue to operate (withdraw sweat from skin) as long as the chemical potential difference between the skin and hydrogel is maintained. In our previous work, we have analyzed the in vitro performance of the patch and studied all its components [14,59,60]. We found that hydrogel equilibrated in 4 M glucose solution functions best in delivering long-term model biomarker collections on paper. Hence, we decided to use a patch with 4 M glucose hydrogel and dry paper for all our human subject studies.

### 3.2. Sampling of Sweat Lactate Using Different Pumping Materials in the Patch

We characterized the efficiency of three different pumping sources in our patch for sampling sweat lactate from the human skin: 4 M glucose hydrogel, PBS hydrogel, and a PDMS hydrogel substitute disk. The three osmolytes were chosen since they have varying levels of osmotic strength and are benign to the human skin upon contact. The 4 M glucose hydrogel has the highest osmotic strength. The PBS hydrogel is isotonic with sweat, while the PDMS disk is inert and has no osmotic action. We embedded each of these materials in the patch and estimated the amount of lactate that they sample on paper under five different physiological conditions: rest (NEX), medium-intensity exercise (MEX, 60–70% of maximum heart rate), post-medium-intensity exercise (PMEX), high-intensity exercise (HEX, >85 % maximum heart rate), and post-high-intensity exercise (PHEX) (Figure S1). The base lactate level under each physiological condition was measured by mechanically rubbing the pad of the paper channel on skin (without hydrogel). This method of lactate collection was referred as the “dry rub” test. Such a comprehensive study would provide a better understanding regarding how effectively lactate is sampled via osmosis under varying physiological conditions, unlike our previous work, where we only used the 4 M glucose hydrogel patch to sample lactate during rest and MEX condition. The exercise protocol for the human trials and the calibration plot of the lactate assay protocol is presented in the Supplementary Information (Figures S2 and S3). The methodology of calibration has been reported in our previous paper [14]. The sensitivity and LOD of our colorimetric lactate assay are 0.18 a.u./mM and 750 µM, respectively.

**Figure 2:**
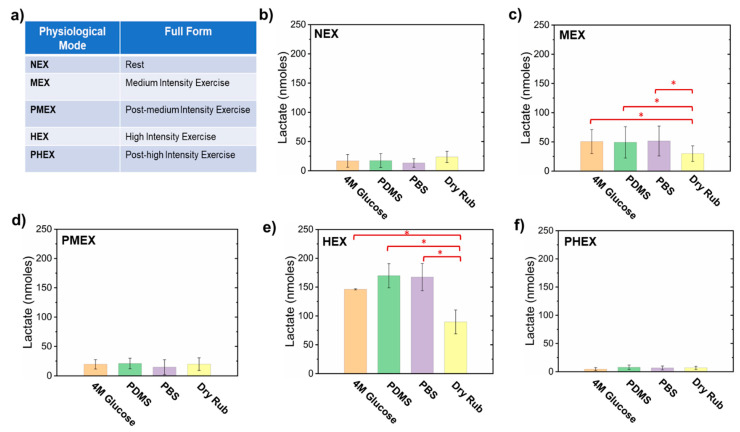
Plots presenting a comparative analysis of the sampled average sweat lactate quantity with different pumping materials in the patch under varying physiological conditions. (**a**) Table highlighting the full forms of all the physiological conditions in which the human subjects were tested. Plot showing the amount of lactate sampled during (**b**) NEX trial, (**c**) MEX trial, (**d**) PMEX trial, (**e**) HEX trial, and (**f**) PHEX trial. These plots show that all pumping materials in the patch can sample lactate on paper. Error bars denote the standard deviation obtained from all subjects. * *p* < 0.05.

**Figure 3:**
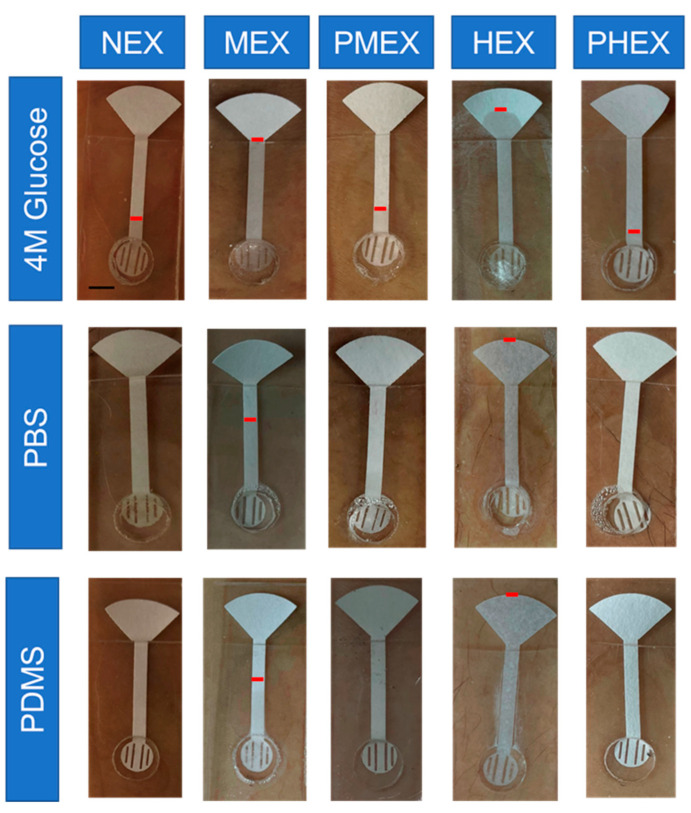
Optical images of the patch on skin showing the levels of sweat collection on the paper channel of patches with different pumping materials under different physiological conditions. The red mark on the paper channel indicates the position of sweat after each trial. Scale bar = 5 mm.

Figure 2 and Figure 3 present a comparative analysis of sweat lactate and volume intake by different pumping materials in the patch under varying physiological conditions, respectively. Figure 2a summarizes the set of all the physiological conditions in which the subjects were tested. After 2 h of rest (NEX) trials, the 4 M glucose hydrogel, PDMS disk, and the PBS hydrogel patch collected ≈17 ± 11 nmoles, ≈17.5 ± 12 nmoles, and ≈13.3 ± 7.5 nmoles of lactate, respectively. The dry rub test resulted in ≈23.63 ± 9.5 nmoles of lactate on paper after 2 h (Figure 2b). The results show that all three pumping materials in the patch can sample lactate from the skin during rest. The 4 M glucose hydrogel patch shows visible fluid flow on the paper channel (Figure 2b and Figure 3). In contrast, the PBS hydrogel patch did not show any visible fluid collection but did collect lactate on paper (Figure 3). As PBS hydrogel is isotonic to sweat, it is evident that the lactate on paper originates from the natural lactate generation rate of the body (≈0.3–1.3 nmole.cm^−2^ min ^−1^) [36]. The same reasoning also holds for the PDMS disk patch (Figure 2b and Figure 3). The lactate readouts of all three pumping materials matched well with the dry rub test (Figure 2b). This justifies that lactate readout of our patch matches well with base lactate levels after 2 h and shows that it can function efficiently under low sweating conditions.

The patches were also tested under conditions of medium-intensity exercise (MEX). The 4 M glucose hydrogel, PDMS disk, and PBS hydrogel patches sampled ≈50.55 ± 20.6 nmoles, ≈49.32 ± 26.8 nmoles, and ≈51.56 ± 25.61 nmoles of lactate, respectively, after 1 h of exercise. The dry rub test resulted in ≈30 ± 13.4 nmoles of lactate collection on paper after 1 h (Figure 2c). The amounts of lactate sampled by all three pumping materials were significantly larger (*p* < 0.05) than the NEX trial results and dry rub test during MEX. All three patch types also showed greater sweat collection on paper than NEX trials.

Exercise increases the energy demand of the body. A part of this high-energy demand is compensated via anaerobic glycolysis, which leads to the generation of lactate in sweat [1,2,3,13,14]. Our patch was able to detect this increase with all three pumping materials. Exercise also leads to “active sweat” generation. Hence, lactate sampled by a 4 M glucose patch is released via both osmosis and active sweat under exercise. The release of active sweat makes the 4 M glucose patch collect more lactate than the dry rub test. PBS hydrogel and PDMS disk patches show elevated lactate levels solely due to the inflow of active sweat, since they do not create osmotic gradients with inherent fluid withdrawing power.

The patches were tested under post-medium intensity exercise (PMEX) conditions for 2 h. The 4 M glucose hydrogel, PDMS disk, and PBS hydrogel collected samples containing ≈19.50 ± 8.0 nmoles, ≈21.0 ± 9.0 nmoles, and ≈14.6 ± 12.6 nmoles of lactate, respectively. The dry rub test resulted in ≈19.8 ± 10.8 nmoles of lactate on paper after 2 h (Figure 2d). The 4 M glucose patch showed visible fluid flow on paper, while the PBS hydrogel patch and PDMS patch did not sample any fluid on paper. During PMEX, the aerobic glycolysis rate increases, and the lactate in sweat gets converted to pyruvate [2]. Hence, the amount of lactate in sweat decreases as the body cools down after exercise. Our patch was able to detect this change with all three pumping materials. Additionally, similar lactate readouts with the NEX trials also indicate that base lactate levels were reached within 2 h after exercise completion in all subjects.

High-intensity exercise (HEX) trials were also conducted with all three patch types. The 4 M glucose hydrogel, PDMS disk, and PBS hydrogel sampled ≈143.76 ± 10.7 nmoles, ≈170.30 ± 14.9 nmoles, and ≈168.10 ± 16.8 nmoles of lactate, respectively. The dry rub test resulted in ≈89.30 ± 14.6 nmoles of lactate on paper after 30 min (Figure 2e). All three patch types sampled ≈5 times larger sweat volume on paper than MEX trials. The amounts of lactate sampled by all three pumping materials and dry rub were significantly larger (*p* < 0.05) than the lactate sampled during MEX trials. Additionally, the lactate readouts of all three pumping materials were significantly higher than the readout of the dry rub test. HEX rapidly induces anaerobic glycolysis and ramps up the lactate levels in sweat [2,13,17]. HEX also leads to greater active sweat release than MEX, which makes all three types of patches sample larger samples of lactate than the dry rub test. This confirms that the amount of lactate in sweat depends on the sweat rate [1,17,18].

The patches were next tested under PHEX conditions for 2 h after the MEX trials. The 4 M glucose hydrogel, PDMS disk, and PBS hydrogel sampled ≈5.05 ± 2.1 nmoles, ≈9.45 ± 3.3 nmoles, and ≈10.09 ± 4.7 nmoles of lactate, respectively. The dry rub test resulted in ≈6.03 ± 5.8 nmoles of lactate on paper after 2 h (Figure 2f). All three patches and dry rub tests showed significantly lower lactate levels (*p* < 0.05) than HEX and PMEX trials. The 4 M glucose patch showed visible fluid flow on paper, while the PBS hydrogel patch and PDMS patch did not sample any fluid on paper (Figure 3). Significantly lower levels of lactate than HEX trials prove that most of the lactate in sweat had been converted to pyruvate within 2 h. Moreover, significantly lower levels of lactate than PMEX trials show that the rate of aerobic metabolism (conversion of lactate to pyruvate) during PHEX is higher than PMEX. Hence, less lactate appears in sweat during the PHEX trials. Our patch was able to detect this change with all three pumping materials. Additionally, similar lactate readouts with the NEX trials also prove that base lactate levels were also reached within 2 h.

### 3.3. Working Mechanism of Different Pumping Materials on-skin toward the Sampling of Sweat Lactate

**Figure 4:**
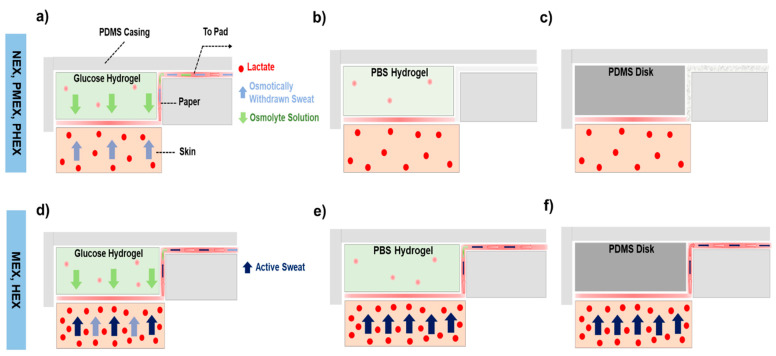
Cross-sectional schematics of sweat lactate transport with different pumping materials at the interface with the skin. The pumping materials include (**a**) a 4 M glucose hydrogel patch, (**b**) a PBS hydrogel patch, and (**c**) a PDMS disk patch during HEX, PMEX, and PHEX trials. Schematic showing the sweat lactate transport in (**d**) a 4 M glucose hydrogel patch, (**e**) a PBS hydrogel patch, and (**f**) a PDMS disk patch during MEX and HEX trials. These schematics show that the osmotic sampling of lactate occurs only in a 4 M glucose hydrogel patch. The PBS hydrogel patch and PDMS disk patch sample lactate from active sweat.

We interpret the transport effects of each pumping media based on the amount of lactate withdrawn by them through the paper–skin interface. Figure 4a highlights how a 4 M glucose hydrogel facilitates lactate withdrawal from skin during NEX, PMEX, and PHEX trials. Once the hydrogel interfaces the skin, osmosis is initiated due to the chemical potential difference between the hydrogel and sweat inside the skin. The glucose solution (from the hydrogel) and the osmotically withdrawn sweat and lactate (from skin) collectively appear in the form of a colorless liquid that wicks onto the rectangular section of the paper channel over time. Since no active sweat is released at rest, the lactate registered on paper is derived completely from the osmotically withdrawn sweat (Figure 2b,d,f) [14]. The PBS hydrogel is isotonic with sweat and has no fluid withdrawing power. Hence, the patch does not show any visible sweat collection at the paper-skin interface (Figure 4b). However, the paper registers a considerable amount of lactate from the skin (Figure 2b,d,f). This lactate amount is an outcome of the wet paper absorbing some of the naturally generated lactate from the human body [14]. The PDMS disk collects lactate on the paper in contact with skin but without any traces of fluid on the paper channel (Figure 2b,d,f and Figure 4c). The absence of fluid on the paper channel is expected, since PDMS has no intrinsic fluid-withdrawing capacity. The lactate appears completely due to its natural production rate in the body.

We also interpret schematically the on-skin action of the pumping materials during MEX and HEX trials. The 4 M glucose hydrogel patch shows greater fluid and lactate collection on the paper channel than the NEX trials (Figure 2c,e). The additional fluid flow arises due to the inflow of “active” sweat in the patch (Figure 4d). Lactate concentration in the active sweat increases with exercise. Hence, the rise in the amount of lactate content on paper is a direct result of active sweat inflow [1,17,18]. The PBS hydrogel also shows greater fluid and lactate collection on paper than the NEX trials (Figure 2c,e). Since a PBS hydrogel has no fluid withdrawing power, the additional fluid and lactate on paper is derived from the inflow of active sweat (Figure 4e). The same collection mechanism also holds for a PDMS disk patch (Figure 2c,e and Figure 4f).

We also evaluated the amount of lactate sampled by the 4 M glucose patch via osmosis under different physiological conditions. This patch selection was made as 4 M glucose hydrogels possessed the highest (in comparison to PBS hydrogel and PDMS disk) osmotic strength. The efficiency of the 4 M glucose hydrogel toward lactate withdrawal via osmosis is expressed in terms of “osmotic contribution (OC)”, which is calculated as:

(1) $$\left(\frac{n_{HG}-n_{PBS}}{n_{PBS}}\right)\times 100$$

where $n_{HG}$ is the moles of lactate sampled by the 4 M glucose hydrogel and $n_{PBS}$ is the moles of lactate sampled by the PBS hydrogel.

**Figure 5:**
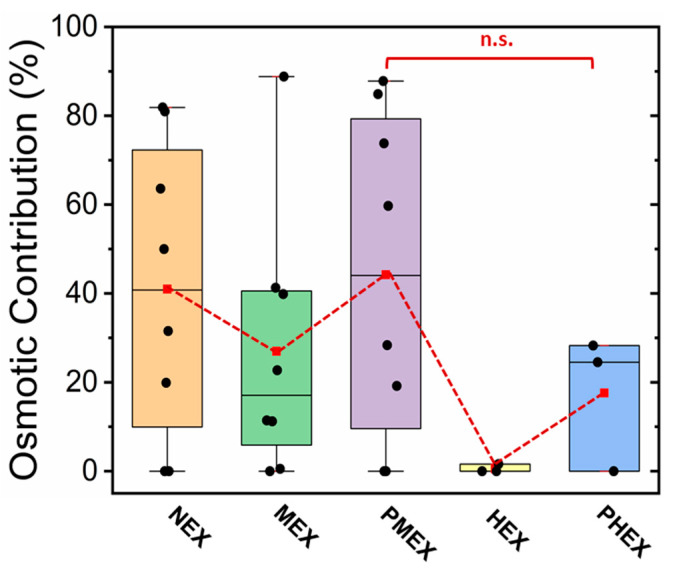
Plot showing the effectiveness of the 4 M glucose hydrogel in facilitating lactate withdrawal via osmosis under different physiological conditions. Each dot denotes data from one subject. The dashed line connects the mean values to guide the eye.

The 4 M glucose hydrogel patch sampled on average ≈40% (NEX), ≈25% (MEX), ≈45% (PMEX), ≈0.5% (HEX), and ≈20% (PHEX) more lactate than a PBS hydrogel patch. The observed variation in the contributions under each condition can be attributed to the difference in the rate of lactate metabolism and to the small differences between the rates of lactate generation by each forearm in each subject (Figure 5).

Lactate collection via osmosis dominates during NEX, PMEX, and PHEX trials due to the absence of active sweat inflow in the paper channel. MEX and HEX trials lead to the active inflow of sweat in the patch, which increases the lactate content on paper. Moreover, HEX leads to larger sweat and lactate collection than MEX but samples lactate the least via osmosis. Hence, the higher the inflow of active sweat, the less the lactate collection via osmosis. Overall, our wearable patch functions by two concurrent mechanisms for sweat lactate withdrawal: (a) osmosis at low sweat rates and (b) active sweat for medium to high sweat rates.

### 3.4. Relationship between Sweat Lactate and Sweat Volume

**Figure 6:**
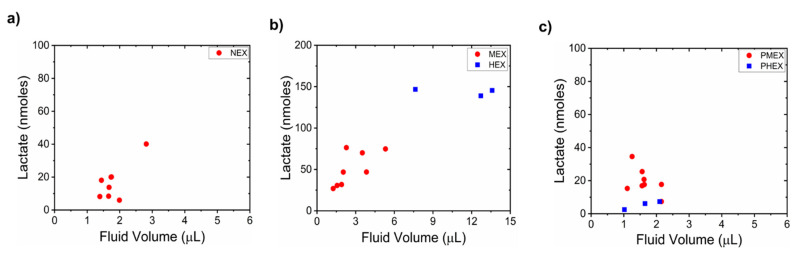
Relationship between sampled sweat lactate quantity (under different physiological conditions) and sweat volume on paper for a 4 M glucose hydrogel patch. Plots showing how sweat lactate depends on sweat volume during (**a**) NEX trial, (**b**) MEX and HEX trials, and (**c**) PMEX and PHEX trials. Each dot denotes data from one subject.

We investigated how the sweat lactate content depends on the sampled sweat volume on paper under all five physiological conditions. This helped us to understand the dependency of sweat lactate on the sweat rate. We analyzed this only for the 4 M glucose hydrogel patch for two reasons: (a) 4 M glucose hydrogel has the highest osmotic strength. Hence, its pumping activity was our major focus of interest. (b) The PBS hydrogel patch did not result in sweat collection on paper during NEX, PMEX, and PHEX trials. Hence, calculation of lactate concentration was not always possible for a PBS hydrogel patch. The 4 M glucose hydrogel sampled ≈1.8 ± 0.4 µL (NEX), ≈2.7 ± 1.3 µL (MEX), and ≈1.6 ± 0.3 µL (PMEX), ≈11.3 ± 3.2 µL (HEX), and ≈1.60 ± 0.5 µL (PHEX) of fluid on paper. During NEX, MEX, HEX, and PHEX trials, the amount of collected sweat lactate increased with the fluid volume on paper (Figure 6a–c). However, we observed an opposite trend during the PMEX trials (Figure 6c).

The 4 M glucose hydrogel produced an average sweat withdrawal rate (SWR) of 0.03 µL.cm ^−2^.min ^−1^, which corresponded to an average sweat volume of ≈1.8 µL after 2 h of testing under NEX. The sampled lactate quantity and sweat volume are in agreement with the results reported earlier by Bhide et al. [58]. A good correlation (Pearson’s correlation coefficient, r > 0.7) existed between the sweat lactate amount and fluid volume on paper. This shows that the amount of moles of lactate varies proportionally with sweat rate at rest (Figure 6a). Hence, osmosis leads to joint sweat and lactate withdrawal during NEX trials (Figure 4a and Figure 5).

The 4 M glucose hydrogel produced an SWR of ≈0.10 µL.cm ^−2^.min ^−1^ and ≈0.75 µL.cm ^−2^.min ^−1^ during MEX and HEX trials, respectively. Such sweat rates are in line with the literature [17,61]. Both sweat and lactate amounts on paper increased during MEX and HEX trials, with HEX collecting the highest amount of lactate (Figure 6b). The observed scattering between the data points is a result of the difference in sweat generation rates of each subject. Around ≈1–1.5 µL, and ≈7–8 µL of additional sweat flows into the paper during MEX and HEX trials, respectively, in comparison to the NEX trials. This additional fluid is active sweat resulting from exercise (Figure 3 and Figure 4d). A good correlation (r > 0.5) between the sampled lactate amount and sweat volume again proves that sweat lactate amount (moles) is a function of sweat rate [1,17,18]. The 4 M glucose hydrogel produced an SWR of ≈0.03 µL.cm ^−2^.min ^−1^ during both PMEX and PHEX trials. Such an SWR is similar to the NEX trials. The lactate amount possesses a negative correlation (r < 0) with the sampled sweat volume on paper during PMEX trials (Figure 6c). As per the review of Bakker et al., this happens since lactate gets converted to pyruvate as the body cools down after exercise [2]. As a result, the amount of lactate in sweat decreases. A positive correlation existed during PHEX trials due to the extremely low magnitude of lactate amount (with respect to PMEX) and sweat volume.

### 3.5. Estimation of Sweat Lactate Concentration and Its Relationship with Blood Lactate

**Figure 7:**
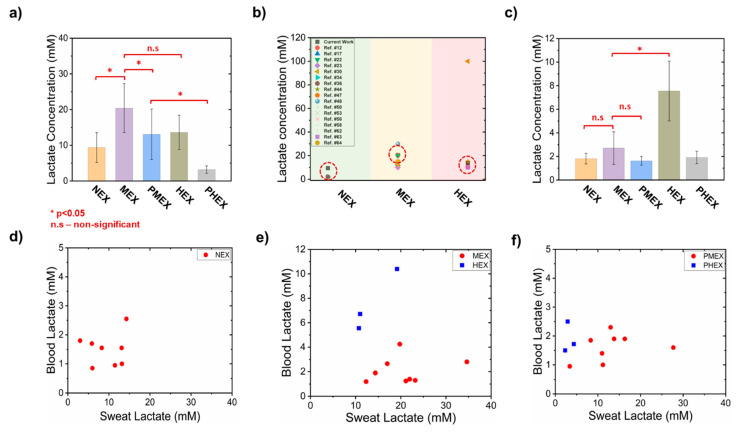
Comparative analysis of sweat lactate concentration readout of 4 M glucose hydrogel patch and blood lactate concentration. (**a**) Plot showing the estimated sweat lactate concentration in a 4 M glucose hydrogel patch under different physiological conditions. (**b**) Plot presenting a comparative analysis of the lactate concentration readout from our patch (dotted circular box) to the lactate concentration data from previously published lactate studies. (**c**) Plot showing the measured blood lactate concentration under different physiological conditions. Relationship of sweat and blood lactate concentration during (**d**) NEX trial, (**e**) MEX and HEX trials, and (**f**) PMEX and PHEX trials. * denotes *p* < 0.05. Each dot denotes data from one subject. These plots elucidate the correlation between blood and sweat lactate levels under different physiological conditions. The error bars denote standard deviation from all subjects.

The amount of sampled lactate and sweat volume on paper were used to calculate the lactate concentration for the 4 M glucose hydrogel patch. The 4 M glucose hydrogel initiates the osmotic withdrawal of sweat upon contact with the skin at a lactate extraction rate (LER) (from skin) of ≈0.58 nmole.cm ^−2^. min ^−1^ (NEX), ≈3.21 nmole.cm ^−2^. min ^−1^ (MEX), ≈0.68 nmole.cm ^−2^. min ^−1^ (PMEX), ≈20.30 nmole.cm ^−2^. min ^−1^ (HEX), and ≈0.16 nmole.cm ^−2^. min ^−1^ (PMEX) (assuming constant sweat generation rate). This corresponded to an average sweat lactate concentration of ≈9.38 mM (NEX), 19.30 mM (MEX), 13.08 mM (PMEX), 13.62 mM (HEX), and 3.18 mM (PHEX) (Figure 7a).

The sweat lactate concentration during NEX trial is considered low, as it falls toward the lower end of the normal human sweat lactate concentration range [6,14,36,38]. Lactate concentration during MEX trials was significantly larger (*p* < 0.05) than NEX trials. This happens because (a) exercise induces anaerobic metabolism, which leads to the appearance of lactate in sweat, and (b) exercise causes greater sweat release, which proportionally releases more lactate (Figure 6b). Lactate concentration during PMEX was significantly lower (*p* < 0.05) than that during MEX, since lactate gets converted to pyruvate as the body cools down after exercise. Additionally, this also suggests that base lactate levels were reached within two hours (*p* > 0.05, with respect to NEX). The difference between the lactate concentrations during MEX and HEX trials were insignificant (*p* > 0.05). Although HEX trials lead to greater amount of lactate generation, a higher volume of sweat release slightly decreases the lactate concentration due to dilution (Figure 2e, Figure 3, Figure 6b and Figure 7a) [18,61]. Hence, as the lactate concentration peaks during MEX, we believe that it is the facile physiological zone for estimating the maximum sweat lactate concentration (Figure 7a,b). Sweat lactate concentrations during PHEX trials were significantly lower than the PMEX trials (*p* < 0.05) due to significant lower lactate generation (Figure 2f). This confirms the following two outcomes: (a) the rate of disappearance of lactate from sweat during PHEX trials is faster than PMEX trials, and it happens within 2 h after HEX termination, and (b) two hours is a sufficient time period to attain the base lactate level. Moreover, the lactate concentration readouts of the 4 M glucose hydrogel patches during NEX, MEX, and HEX trials match well with the lactate concentration readouts of existing devices, which were reported under similar exertion levels (rest, medium-intensity exercise, and high-intensity exercise) (Figure 6b) [1,5,12,14,17,23,30,34,36,42,43,44,47,48,50,53,56,58,61,62,63,64]. This validates the efficient functioning of our patch under all physiological conditions.

We also measured blood lactate levels and investigated its correlation to sweat lactate under all five physiological conditions (Figure 7c–f). A poor correlation between sweat and blood lactate levels was recorded during NEX trials (Figure 7d). This is expected due to the lack of physical exertion. Hence, as suggested by Anderson et al., the lactate in sweat and blood during NEX is mainly derived from the sweat glands above type I muscle fibers [65]. Type I muscle fibers generate energy mainly by aerobic metabolism. The measured average blood lactate concentration also matches well with what Alam et al. have reported in their review [3]. Blood lactate concentration during MEX trials remained low and similar to NEX trials (*p* > 0.05) (Figure 7c). Hence, MEX does not affect the blood lactate levels. This also results in a poor correlation between blood and sweat lactate levels (Figure 7e). Such trends are expected, since we operate below the lactate threshold (LT) of the human subjects during MEX trials [61]. Goodwin et al. have shown that blood lactate increases exponentially beyond LT [21]. The data also established the source of lactate origination to be mainly the sweat glands above the type IIa muscles and not blood [65]. Type IIa muscle fibers generate energy under both aerobic and anaerobic metabolism. The poor correlation between blood and sweat lactate levels during PMEX exists due to the absence of physical exertion and glycolysis in type I muscle fibers (Figure 7f).

HEX causes a significant rise in the blood lactate levels (*p* < 0.05, with respect to MEX) (Figure 7c). Consequently, the ratio between sweat and blood lactate concentration decreases (with respect to MEX). This results mainly from the following two reasons: (a) HEX makes the subjects exceed their LT (>85% of maximum heart rate) [21]. This causes the lactate to partition into blood from the type IIb muscle fibers. Anderson et al. have shown that the type IIb muscle fibers generate energy purely via anaerobic glycolysis [65]. (b) A drop in the HCO_3_^−^ ion concentration and pH in plasma leads to an inflow of lactate in blood to maintain the acid–base equilibrium [19]. Overall, a prolonged high blood lactate concentration (> 3 mM) induces faster fatigue and is physiologically challenging [65,66,67]. Hence, individuals can be recognized as being “fit” if they can delay reaching LT or show low blood lactate levels even under HEX [65]. A strong correlation exists between blood and sweat lactate levels during HEX (Figure 7e). The correlation exists due to higher blood lactate levels than those in the MEX trial. Such correlations have been reported by Karpova et al. previously [1]. A poor correlation exists between blood and sweat lactate levels during PHEX trials (Figure 7f) due to the absence of high physical exertion.

## 4. Conclusions

We report here the on-skin functioning of a non-invasive sweat sampling patch that operates under novel osmotic extraction principles. The patch uses a hydrogel disk infused with a solute to raise its chemical potential difference with respect to sweat in the skin. As a result, the hydrogel directly withdraws the sweat from the skin via osmosis and without the necessity of active perspiration. The extracted sweat is transported via a paper channel and evaporated at a pad to maintain the continuous inflow of sweat and its associated biomarkers. Since osmosis is a colligative property inherent to the system, the patch does not require any external power source for long-term operation. The patch will continue to withdraw sweat as long as the chemical potential difference sustains between the hydrogel and sweat. Moreover, this patch design is flexible, non-invasive, and can be comfortably worn on the skin.

We tested the patch with three different pumping media under five different physiological conditions for sweat lactate quantification. Results show that all three pumping media sample lactate on paper during rest, exercise, and post-exercise trials. Lactate was majorly withdrawn by the glucose hydrogel via osmosis when the body remained exertion-free (NEX). The PBS hydrogel and PDMS disk patches sample lactate on paper due to the natural lactate generation rate of the body during NEX trials. The moles of sweat lactate collected increase with exercise intensity and decrease when the body cools down. During exertion (MEX and HEX), lactate is derived primarily from the inflow of active sweat in all three patch systems. Hence, the amount of sweat lactate captured is proportional to the sweat rate. The calculation of sweat lactate concentration was only possible for the glucose hydrogel patch, as there was considerable sweat collection on the paper. The lactate concentration measured by the glucose hydrogel patch correlated well with the lactate concentration readouts of other sensors, which were tested under similar exertion levels. Sweat lactate concentration during HEX remained lower than MEX. This occurs because the rate of sweat production (volume/time) is greater than the rate of lactate generation (moles/time) in sweat during HEX, which eventually leads to dilution and a lower lactate concentration (moles/volume). Hence, sweat lactate concentration should be measured under MEX as it peaks during this physiological state. Blood lactate concentration stayed low and did not show a good correlation with sweat lactate concentration during NEX, MEX, PMEX, and PHEX trials. This occurred due to the absence of physical exertion. In contrast, the blood lactate concentration increased and showed a good correlation with sweat lactate concentration during HEX. This occurred since all subjects surpassed their LT during HEX. The variation in sweat and blood lactate concentration levels under different physiological conditions is presumably an outcome of varying glycolysis rates at different muscle fiber sites.

Overall, our wearable osmotic sweat sampling patch appears to have big potential in facilitating continuous sweat collection for hours and delivering useful health information about human lactate trends under varying physiological conditions. Despite the on-skin studies, our patch requires post-processing measures for estimating the amount of lactate in sweat. After testing, the rectangular strip needs to be cut out and subjected to an assay for lactate determination. Hence, the current version of the patch is not an optimized solution for device operation, since an ideal wearable should deliver continuous monitoring and real-time data generation. Our group is working toward extending the current version of the patch to a continuous, real-time sweat sensing platform for lactate by incorporating enzymatic electrochemical sensors. In addition to lactate, the prototype reported here can be used to sample multiple other sweat based biomarkers as well. We are also working toward transforming our current patch to a lateral flow assay (LFA) platform for cortisol and potassium detection in sweat. We believe that such a prototype, capable of functioning with multiple sensing principles (colorimetric, electrochemical) will provide a better, long-term understanding about the metabolism of different sweat-based biomarkers under both at home and clinical settings.

## Supplementary Materials

The following are available online at https://www.mdpi.com/article/10.3390/mi12121513/s1. Figure S1: Schematic illustration of the human trial protocol. 4 M glucose hydrogel, PBS hydrogel, and PDMS disk patches were tested on skin during rest, exercise, and post-exercise conditions for sweat lactate estimation. The dry rub test estimates the base lactate level during each physiological stage. Figure S2: Rotational speed of the cycle ergometer and exercise intensity range during human trials. Figure S3: Calibration plot of normalized intensity vs. lactate concentration.
