# Supplementary material for: Osmotically Enabled Wearable Patch for Sweat Harvesting and Lactate Quantification
Source: Micromachines (Basel). 2021 Dec 4;12(12):1513. doi: 10.3390/mi12121513 (PMC8705979; doi:10.3390/mi12121513)
Supplement: Supplementary file 1 [file micromachines-12-01513-s001.zip › micromachines-1461793-supplementary.pdf]

Article

Osmotically Enabled Wearable Patch for Sweat Harvesting and Lactate Quantification

Tamoghna Saha, Jennifer Fang, Sneha Mukherjee and Charles T. Knisely, Michael D. Dickey and Orlin D. Velev

Supplementary note 1: On-skin testing procedure with the osmotic patch

The subjects were initially made to sit and rest. The forearm regions of both hands were then cleaned with alcohol wipes and de-ionized (DI) water to remove any residual lactate. Three skin patches (each with a 4M glucose hydrogel, PBS hydrogel, and PDMS disk) were interfaced to the washed part of each forearm using a Velcro strap for two hours. The subjects remained seated during the non-exercise phase (NEX) and refrained from consumption of food and drinks. After 2 hours, the patches were taken off, their paper strips were removed, a dry rub test was performed by rubbing a new paper strip on the skin, and blood lactate was measured. The wet portion (4M glucose hydrogel patch) and the dry circular end (PBS hydrogel, and PDMS disk patches) of the paper channel were cut out and reserved for further analysis. This concluded the NEX trial.

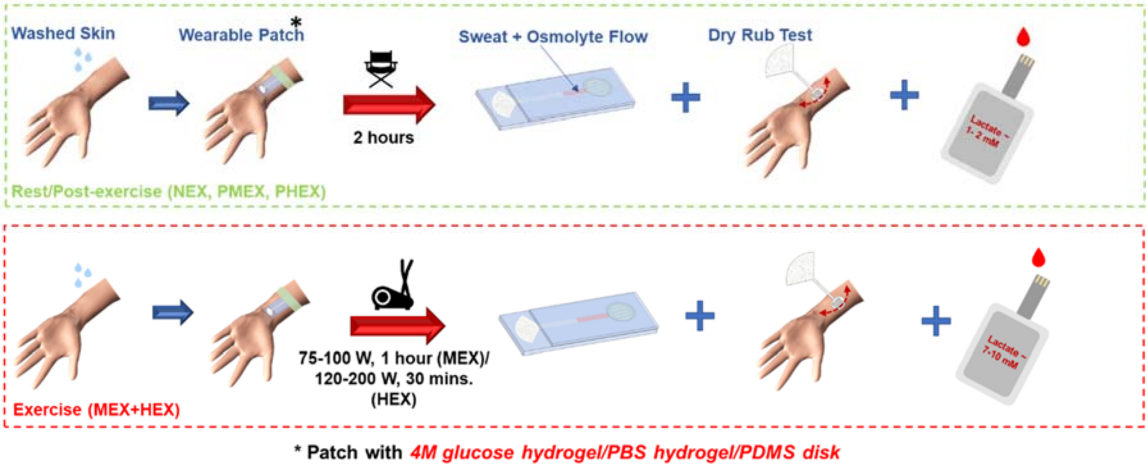

Figure S1. Schematic illustration of the human trial protocol. 4 M glucose hydrogel, PBS hydrogel, and PDMS disk patches were tested on skin during rest, exercise, and post-exercise conditions for sweat lactate estimation. The dry rub test estimates the base lactate level during each physiological stage.

The subjects were then taken to an exercising room where their forearms were cleaned again with alcohol wipes and DI water. Three patches (each with 4M glucose hydrogel, PBS hydrogel, and PDMS disk) were again interfaced on each forearm. The subjects exercised on a cycle ergometer (Life Fitness) under ambient conditions for an hour during medium exercise intensity (MEX) trials, and for 30 mins during high exercise intensity (HEX) trials. MEX trials involved 5 minutes of warmup (50-60 W at 60 rpm), followed by 20 minutes of constant load exercise at medium intensity (75-100 W at 75 rpm) and a final 5 minutes of cooling down. This cycle was repeated for another 30 minutes. After an hour of testing, the patches were taken off, their paper strips were removed, a dry rub test was performed by rubbing a new paper strip on the skin, and blood lactate was measured. The wet portion (4M glucose hydrogel patch) and the dry circular end (PBS hydrogel, and PDMS disk patches) of the paper channel were cut out and reserved for further analysis. This concluded the MEX trial.

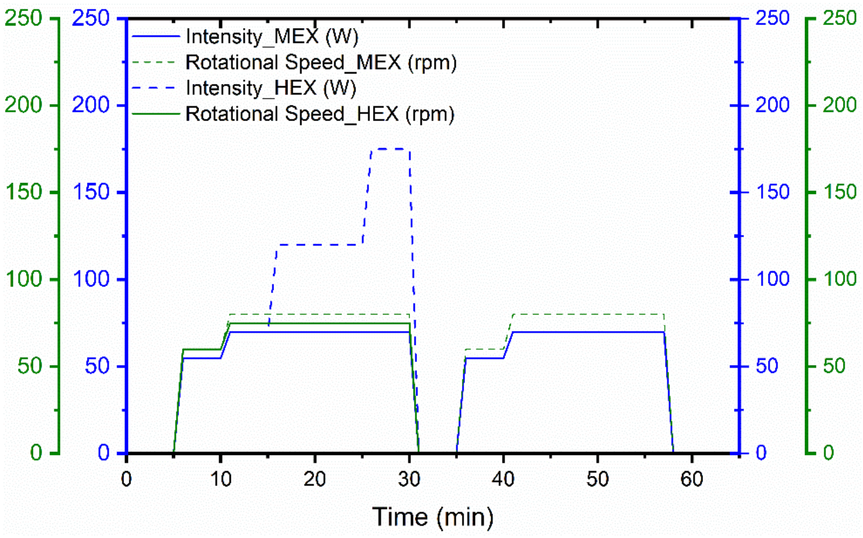

Figure S2. Rotational speed of the cycle ergometer and exercise intensity range during human trials.

HEX trials involved 5 minutes of warmup (50-60 W at 60 rpm), followed by 5 minutes of constant load exercise at 100 W and 75 rpm. The intensity was then increased to high intensity (100-150 W for 10 mins with 75 rpm), followed by 5 minutes at 150-200 W and 75 rpm. Exercise was then terminated, and the subjects rested for 5 minutes. After completion of each trial, the patches were taken off, paper strips were cut and reserved for analysis, a dry rub test was conducted, and blood lactate was measured. This concluded the HEX trial.

Post medium exercise (PMEX) and post high exercise (PHEX) trials were conducted in a similar way as the NEX trials. The paper strips after each test were analyzed using a commercial colorimetric lactate assay (Lactate dehydrogenase enzyme based) with our previously developed protocol [1].

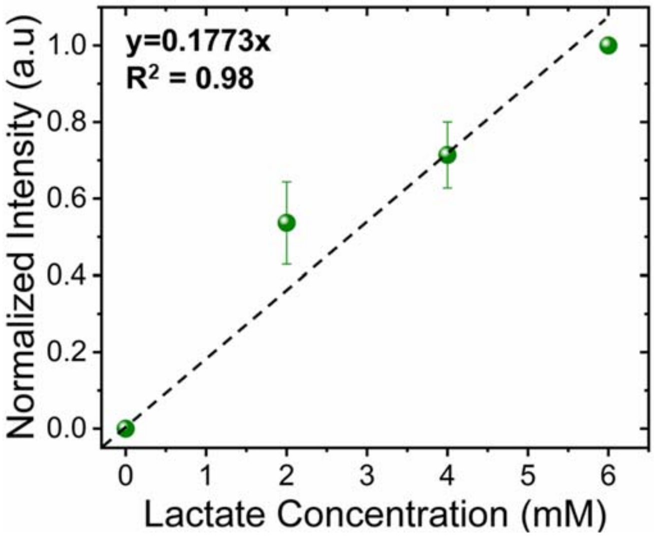

Figure S3. Calibration plot of normalized intensity vs. lactate concentration.

References:

1. Saha, T.; Fang, J.; Mukherjee, S.; Dickey, M. D.; Velev, O. D. Wearable Osmotic-Capillary Patch for Prolonged Sweat Harvesting and Sensing. *ACS Appl. Mater. Interfaces* **2021**, *13*, 8071–8081.
